# Construction and Operation of a Respiration Chamber of the Head-Box Type for Methane Measurement from Cattle

**DOI:** 10.3390/ani10020227

**Published:** 2020-01-31

**Authors:** Octavio Alonso Castelán Ortega, Paulina Elizabeth Pedraza Beltrán, Gloria Stefanny Hernández Pineda, Mohammed Benaouda, Manuel González Ronquillo, Luisa T Molina, Juan Carlos Ku Vera, Hugo Daniel Montelongo Pérez, María Fernanda Vázquez Carrillo

**Affiliations:** 1 Facultad de Medicina Veterinaria y Zootecnia, Instituto Literario 100, Universidad Autónoma del Estado de México, Colonia Centro, 50000 Toluca, Estado de México, Mexico; pb_eli@yahoo.com.mx (P.E.P.B.); stefyhernandez112@hotmail.com (G.S.H.P.); mohammed.ben-aouda@inra.fr (M.B.); mrg@uaemex.mx (M.G.R.); hugo_as159@hotmail.com (H.D.M.P.); mvz.mafervazquez@gmail.com (M.F.V.C.); 2 Molina Center for Strategic Studies in Energy and the Environment, La Jolla, CA 92037, USA; ltmolina@mit.edu; 3 Facultad de Medicina Veterinaria y Zootecnia, Universidad Autónoma de Yucatán, Carretera Mérida-Xmatkuil km 15.5, 97100 Mérida, Yucatán, Mexico; kvera@correo.uady.mx

**Keywords:** respirometry system, Ym factor, emission factor, cows, heifers

## Abstract

**Simple Summary:**

The aim of the present work is to describe the construction and operation of a respiration chamber of the head-box type for measuring methane emissions from bovines. Methane is a greenhouse gas 28 times more potent than CO_2_ in its capacity of producing the greenhouse effect and global warming. This gas is produced in considerable amounts by cattle as part of its normal digestion process; approximately 37% of the global anthropogenic methane emissions originate from the livestock industry. Measuring emissions of methane by cattle is necessary for inventory calculation and the evaluation of mitigation policies of this gas. The gold standard technique for measuring methane emissions from cattle is the respiration chamber; however, respiration chambers are expensive pieces of equipment that are not easily available for developing countries. Since a large proportion of the world’s cattle population is in the developing countries, a cheaper option is necessary. A respiration chamber of the head-box type is an option because of its low cost and high accuracy in estimating emissions. This chamber can be used to determine in vivo methane emission factors for those countries that do not have full respiration chambers. It can also be used to conduct experiments to evaluate the anti-methanogenic effects of different compounds.

**Abstract:**

This paper aims to describe the construction and operation of a respiration chamber of the head-box type for methane (CH_4_) measurements in bovines. The system consists of (1) a head box with a stainless steel frame and acrylic walls, floor, and ceiling; (2) a stainless steel feeder; (3) an automatic drinking water bowl; (4) a hood made from reinforced canvas; (5) an infrared (IR) CH_4_ gas analyzer, a mass flow generator, a data-acquisition system; and (6) a steel metabolic box. Six assays were conducted to determine the pure CH_4_ recovery rate of the whole system in order to validate it and comply with standards of chamber operation. The gravimetrical method was used for the recovery test and the recovery rate obtained was 1.04 ± 0.05. Once the system was calibrated, measurements of CH_4_ were conducted using eight animals consisting of four Holstein cows with a live weight of 593.8 ± 51 kg and an average milk yield of 23.3 ± 1.8 kg d^−1^ and four heifers with a live weight of 339 ± 28 kg. The CH_4_ production values were 687 ± 123 and 248 ± 40 L CH_4_ d^−1^ for cows and heifers, respectively. The CH_4_ yield was 19.7 ± 3.4 g and 17.1 ± 3.4 g CH_4_ kg^−1^ of dry matter consumed for cows and heifers, respectively. These results are consistent with those reported in the literature.

## 1. Introduction

Recently, there has been growing global concern regarding the interlinkage of climate change and air quality, thus inspiring many investigations into evaluating the effects of greenhouse gases (GHGs) on the environment. Cattle are responsible for approximately 53% of GHG emissions from agricultural sources, with approximately 87.4 million tons of CO_2_ eq. being emitted worldwide in 2012 [1,2]. A significant portion of environmental GHG emissions derives from the process of rumen fermentation that constitutes a highly specialized type of energy metabolism by strictly anaerobic Archean methanogenic prokaryotes [3] found in the digestive tract of ruminants. It has been estimated that approximately 37% of the world’s CH_4_ emissions are produced by the livestock sector [4]. In recent years, interest in accurately measuring these emissions and developing emission inventories to support GHG-mitigation strategies has increased. On the other hand, the complexity and variation among livestock production systems and the high cost of the analytical equipment make quantifying GHG emissions difficult in developing and underdeveloped countries.

In Mexico, GHG inventories have been carried out since 1997 with contrasting results. The estimated CH_4_ emissions from livestock enteric fermentation were determined using the methodology proposed by the Intergovernmental Panel on Climate Change [5] Tier 1, which is based on the use of default emission factors for different categories of animals. In 2015, the Tier 1 inventory reported by the National Institute of Ecology and Climate Change-Ministry Environment and Natural Resources of Mexico (INECC-SEMARNAT) [6] for methane emissions from the enteric fermentation of cattle in Mexico was of 1710 Gigagrams year^−1^. In contrast, Castelán-Ortega et al. [7] reported an inventory of 2028 Gg year^−1^ by using a mechanistic model to simulate CH_4_ emissions from the enteric fermentation of cattle. Few scientific studies have addressed the CH_4_ emissions from cattle in Mexico, and their results are inconsistent. For example, Ruiz-Suárez and González-Avalos [8] estimated the CH_4_ emissions produced by cattle using a mathematical model, modifying the The Intergovernmental Panel on Climate Change (IPCC) Tier I methodology and calculating the energy consumption based on cattle live weight. They found that, nationwide, dairy cattle produced 288 Gg of CH_4_ in 1995, while INECC-SEMARNAT [6] reported an emission of about 241 Gg year^−1^ also for dairy cattle, despite increased cattle population. In contrast, Rendón-Huerta et al. [9] estimated CH_4_ emissions using the methodologies proposed by IPCC Tier II, the model of Moe and Tyrrell [10], and a mechanistic model (COWPOLL), and found that the CH_4_ emissions were 17.5%, 32.7%, and 69.4%, respectively, lower than those reported by Ruiz-Suárez and González-Avalos [8] for that particular year. Rendón-Huerta et al. [9] predicted that in 2020, CH_4_ production from lactating dairy cows alone in Mexico could increase to 2780 Gg year^−1^.

As described above, substantial disparities persist in the existing inventories, resulting in high uncertainty. This uncertainty can be attributed to the fact that in Mexico, the production of CH_4_ by cattle had not been measured in vivo, and therefore, the in vivo emission factors had not been determined. The high cost of acquiring and constructing the equipment associated with laboratories has impeded the conduction of experiments to determine the emission factors, which are needed to yield better inventories and reduce the degree of uncertainty. Respiration chambers of the head-box type offer the opportunity for measuring CH_4_ emissions but at a fraction of the cost of a full chamber and with similar precision. 

The objective of this study is to describe the design, construction and implementation of a respiration chamber of the head-box type to measure emissions of CH_4_ produced by cattle.

## 2. Materials and Methods

### 2.1. Design and System Components. 

**Figure 1:**
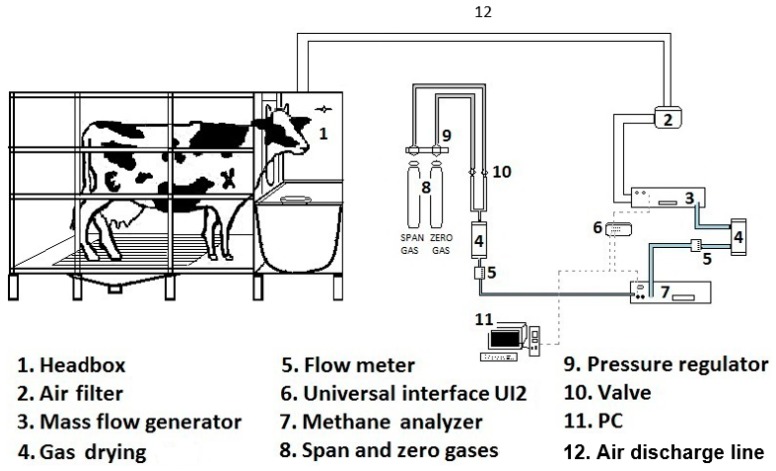
Schematic diagram of the ventilated hood-type open circuit respiration chamber.

**Figure 2:**
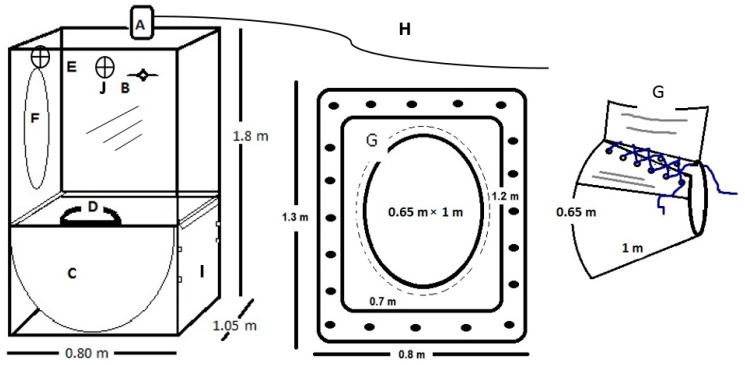
Schematic of the head-box: (**A**) Air outlet connecting to the mass flow generator (FK-500); (**B**) Air inlet valve; (**C**) Feeder; (**D**) Automated drinking water bowl; (**E**) Transparent acrylic walls (0.6 cm thick); (**F**) Hood hole; (**G**) Hood; (**H**) Insulated hose that connects the head-box with the mass flow generator, (**I**) feeder door, (**J**) fans.

The chamber was built at the FMVZ-UAEMex, which is located in Central Mexico (19°5” N and 98.2° W) at an altitude of 2600 m. The design of the chamber was based on Suzuki et al. [11] and Place et al. [12] with modifications to improve the animal’s comfort, the security of the operator and the accuracy of the measurements. For example, of the design included a metabolic cage for cattle that facilitates feces and urine collection for calorimetry and digestibility studies and at the same time guaranteeing the security of the operator, a cage floor covered with non-sliding nonskid and cushioned material to ensure the animal’s welfare, a stainless steel feeder with a semi-cylindrical shape bottom that facilitates the food intake by the animal, a feeder mounted on rails so that it can be slid toward the animal to provide extra food if needed, two fans that keep the animal fresh and ensure full gas mixing within the box, and a smaller head-box than that in Suzuki et al. [11], which helps to prevent leaking of CH_4_. A schematic diagram of the system configuration and the relevant instrumentation is shown in Figure 1. The system consists of the following components:

1. The head-box was designed based on the average head and neck sizes of large breeds of dairy cattle, e.g., Holstein and Brown Swiss cows (Figure 2). The dimensions (width × length × height) of the head-box were 1.05 m × 0.8 m × 1.80 m. The frame of the head-box was made of stainless steel (3.5 cm × 3.5 cm), and the walls, ceiling, and floor were constructed of sheets of transparent acrylic (0.6 cm thick). Acrylic was used to ensure the complete visibility of the animal. The front of the head-box included a removable acrylic door with dimensions of 1.0 m × 0.75 m × 0.50 m, which was sealed by sliding latches on the side of the box and allowing access to the feeder. On the back of the head-box, an oval opening (0.65 m × 1 m) allows the cow’s head to enter the box. A hood was added to the box’s opening (Figure 2G). 

2. The stainless-steel feeder had dimensions of 1.0 m × 0.75 m × 0.50 m and a capacity of approximately 20 kg of forage on a dry matter (DM) basis. The bottom was semi-cylindrical in shape, and the feeder was mounted on rails so that it could be slid toward the animal to provide food. The feeder could be taken out of the head-box without removing the animal from the chamber and the cage (Figure 2C).

3. The automatic drinking water bowl with dimensions of 0.30 mm × 0.25 m × 0.10 m was located on the side wall of the head-box (Figure 2D). The bowl was connected by a 0.5-inch hose to a 450-L water storage tank located 3 m above ground. The tank was installed on a tank stand made of steel providing permanent water supply to the animal. 

4. The hood was manufactured from reinforced cotton fabric and canvas. The part of the hood attached to the head-box was made of reinforced canvas; it was rectangular with dimensions of 0.85 m × 1.30 m (width × height). The part of the hood that went around the animal’s neck was made of cotton fabric; it was oval with dimensions of 0.65 m × 1 m (Figure 2F,G). This hood allowed the animal to move freely while eating, ruminating, or lying down without removing its head from the box. It was designed to fit bovines with live weights ranging from 100 to 700 kg. 

5. Two 30 cm fans (model EE90522G, Bizline, Beijing, China) were mounted on the right and left panels, to keep animals fresh and ensure full gas mixing within the box (Figure 2J).

6. The CH_4_ emission sampling system consisted of the elements shown in Figure 1:

1.7) A dual-wavelength infrared optical bench CH_4_ analyzer (model: MA-10, range 0%–10% and high resolution 0.0001% to 0.01%), barometric pressure (Sable Systems International, Las Vegas, NV, USA) compensated and not sensitive to flow rate changes, which eliminated errors caused by ambient pressure variation.

1.3) A mass flowmeter/generator (MFG) with a range from 50 to 500 L/min and an internal down-sampler with rotameter and a real-time outflow reader (Model: FK-500 Sable Systems International, Las Vegas, NV, USA). This equipment combined a precise mass flow sensor with a rotary pump controlled by an onboard microprocessor. The drive to the pump was modulated under a proportional–integral–derivative controller (PID) to maintain the desired flow rate, already corrected for standard conditions for temperature and pressure. The accuracy was 2% of full scale and the resolution was 1L min^-1^.

1.6) A data-acquisition system (UI2). Model: Universal Interface UI2 also made by Sable Systems International, Las Vegas, NV, USA.

**Figure 3:**
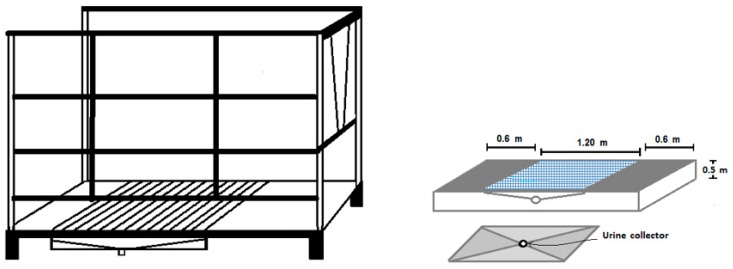
Schematic of the metabolic cage and feces-urine collector.

7. The metabolic cage was constructed of steel tubing with dimensions of 2.44 m × 0.8 m × 1.8 m and a separation of 0.40 m between the tubes constituting the sides and the rear door (Figure 3). The metabolic cage had a front head holder (0.05 m wide) that prevented the animal from removing its head from the head-box. The stainless-steel floor included a tray with dimensions of 1.20 m × 1.05 m; the bottom of the tray was 0.10 m lower than the sides to allow for the collection of urine in a 1-in polyvinyl chloride (PVC) tube.

The floor around the pen was covered with non-sliding nonskid material and cushioned to ensure the animal’s welfare. The access to the cage consisted of a concrete ramp covered with nonskid material (Figure 3).

### 2.2. Operation of the CH_4_-Measurement System to Determine the Production of CH_4_ by Cattle. 

The head-box was connected by the air outlet (Figure 2A) to the MFG by a 1.5-in diameter and 4-m long insulated hose (Figure 2H), which was located at the top center of the head-box. A 1-mm mesh filter located on the air outlet prevented the passage of large particles and flies and a second filter (Figure 1, 2) prevented the passage of dust from the head-box to the mass flow generator. This set-up pulled air through the head-box at a constant flow rate of 500 L min^−1^ and collected a subsample of 0.5 L seg^−1^; which was sent to the CH_4_ analyzer; the rest of the air was automatically discarded through a hose (Figure 1, 12) out of the laboratory building.

The subsample from the MFG passed through a laboratory gas drying unit (Figure 1, 4; model #26800, WA Hammond Drierite, Co. LTD. USA) using a 0.5-mm plastic hose. The airflow then passed through a flowmeter (Figure 1, 5), range 0.15-1 SLPM air (Model VFA-22-BV, Dwyer, Instruments, Inc.) and then to the CH_4_ analyzer at a rate of 0.300 L min^−1^, and the readings generated by the CH_4_ analyzer were sent to a computer by means of a universal interface (UI2) (Figure 1, 6).

### 2.3. Methane Recovery Test and Calibration of the Whole System

This was conducted to determine the CH_4_ recovery rate of the system in order to comply with adequate international standards of chamber operation as suggested by [13]. The gravimetrical method described by Arceo-Castillo et al. [14] was used to conduct the recovery test. Pure CH_4_ gas (calibration gas PRAXAIR, Mexico UN-1971, key ME2.0-D34), was released in different assays. All calculations of methane recovery rates were carried out in the ExpeData software (v.1.9.11; Sable Systems International, Las Vegas, NV, USA). The data from the methane analyzer were recorded and transferred to the computer in real time. In the first five assays, small amounts of methane were released because of the high cost of the CH_4_ standard. In these assays, the gas was blended into the chamber at an average rate of 0.133 g min^−1^ or 7.98 g h^−1^. For the last assay, 28.51 g of pure methane were released inside the respiration chamber at a rate of 0.27 g min^−1^ or 16.2 g h^−1^, which is closer to the CH_4_ emission rate of 20.5 g h^−1^ recorded on the experimental cows. The high cost of pure methane in Mexico prevented us from releasing larger amounts of gas for longer periods of time. The release rate was controlled by an analytical regulator (Praxair ProStar, Platinum, model PRX 417, flow: 0.5 SLPM). The recovery rates were calculated based on the gravimetric volume (L) of methane released into the respiration chamber, a precision balance was used to continuously weigh the CH_4_ cylinder considering a methane density of 0.716 g L^−1^ and the methane volume recovered in the analyzer during the duration of each experimental run. For the calculations, the procedure described by Arceo-Castillo et al. [14] was used as shown in Equations (1)–(3):

(1) $$VCH4L=\frac{Iw-Fw}{D}$$

(2) $$VCH4R=(\frac{CCH4}{100})\times f\times t\times 60$$

where *VCH4L* is the methane volume released in the chamber, *Iw* and *Fw* are the initial and final weights of the cylinder in g, and *D* is the density of methane. *VCH4R* is the methane volume recovered in the chamber, *CCH4* is the average CH_4_ concentration (%) from the start of gas release until the background concentration is reached after closing the methane cylinder, *t* is the total measurement time in *h*, and 60 is the conversion constant in *h*, *f* is the air extraction rate in L min^−1^. So, the recovery rate was determined as shown in Equation (3).

(3) $$\%Rec=\left(\frac{VCH4L}{VCH4R}\right)\times 100$$

Table 1 shows a mean recovery rate of 1.04 ± 0.05 with a recovery range from 0.99 to 1.12. The recovery rates were similar to those reported by [14,15,16], indicating that the operation of the head-box respirometry system is within acceptable international standards. The system was capable of detecting accurately small volumes of methane released, a feature not available in some chambers because methane tends to dilute with the air within the chamber [14].

### 2.4. Analyzer Calibration

Before each assay, two calibrations of the chamber were performed: a zero calibration using high-purity nitrogen (N_2_) (Praxair Inc., Toluca City, Mexico), and a calibration against a reference gas, known as span gas. The N_2_ in the zero calibration was first passed through a drying unit to remove moisture and then through the methane analyzer at a flow rate of 0.3 L min^−1^ until a zero reading. The span calibration was performed using a known CH_4_ concentration gas mixture (1000 ppm of CH_4_ in high-purity N_2_). The span gas passed through the analyzer (0.3 L min^−1^) to obtain a stable reading corresponding to the concentration of CH_4_ in the span gas. (1000 ppm of CH_4_ in high-purity N_2_). The released methane volumes were kept constant by adjusting (10 psi) pressure regulators (Concoa 109–6504) to a controlled flow of 0.2 LPM by means of a flowmeter. The background CH_4_ concentration was verified by injecting ambient air into the analyzer; the air samples were taken with a vacuum pump (PADIIVI.021, APT Instruments, Rochester, NY, USA) at the point of entry of air into the respiration chambers.

### 2.5. Experimental Procedure for the in vivo Measurement of CH_4_ Emissions

All animal-handling procedures used in this study were performed following the guidelines for the care and use of experimental animals and were conducted with the permission of the Committee on Animal Health and Welfare of FMVZ-UAEMex.

### 2.6. Animals and Diet

In the present study, eight animals of the Holstein breed in two age ranges were used: four heifers (12‒13 months) and four cows older than 24 months. One month prior to the start of the experiment, the animals were dewormed and acclimated to the chamber. The cows were at the end of their early lactation and had an average live weight of 593.8 ± 51 kg and an average milk yield of 23.3 ± 1.8 L d^−1^. The average weight of the heifers was 339 ± 28 kg. The two groups were fed a diet consisting of concentrate and corn + alfalfa silage in a 50:50 ratio with ad libitum access to water. The concentrate was composed of 487 g kg^−1^ of corn grain, 200 g kg^−1^ of soybean, 148 g kg^−1^ of canola, 147 g kg^−1^ of wheat bran, and 18 g kg^−1^ of minerals. The cows received 7.5 kg DM d^−1^ of concentrate. The heifers received 1.9 kg DM d^−1^ of concentrate. The chemical compositions of the silage and concentrate are shown in Table 2.

### 2.7. Measurement of Methane Production and Feed Digestibility

Each animal remained in an individual pen for 5 days; the digestibility was measured on Days 4 and 5, whereas CH_4_ production was measured on Day 5 of this period. The animals were weighed at the start of the assay.

Methane production: The CH_4_ emissions were measured for 24 h in each assay. The cows were removed from the chamber for milking at 06:00 and 15:00, and each milking lasted 1.5 h, after which they returned to the chamber to complete 24 h of measurement. The heifers remained in the chamber for 24 h continuously. Every assay started at 10:00, the diet was supplied, the mass flow generator was set at 480 L/min, and the acquisition data system was set to record CH_4_ concentration every second and then the animal entered the chamber. 

Digestibility measurement: The offered diet was weighed daily, and all animals were fed at the same time. The next morning, the orts were removed and weighed to calculate the DM intake (DMI). The samples were collected and kept in a freezer until laboratory analysis. Feces and urine were collected and weighed at the end of the day. A sample of approximately 1 kg of feces was obtained and kept frozen until laboratory analysis. The digestibility of the dry matter (DMD) intake was calculated as shown in Equation (4).

(4) $$DMD\left(\%\right)=\left(\frac{DMI-FAw}{DMI}\right)\times 100$$

where *DMD* = dry mater digestibility, *DMI* = total daily dry matter intake (kg d^−1^), and *FAw* = feces weight (kg DM d^−1^) 

The digestibility of the gross energy intake (GED) was calculated as shown in Equation (5).

(5) $$GED\left(\%\right)=\left(\frac{GEI-GEf}{GEI}\right)\times 100$$

where *GEI* = daily gross energy intake (MJ d^−1^) and *GEf* = gross energy content in feces (MJ d^−1^).

### 2.8. Chemical Analysis of the Feed and Stool

Feces, concentrate, and silage samples were dried in a forced air oven at 60 °C for 72 h, ground, and passed through a 1-mm sieve. The DM and organic matter (OM) contents were determined according to the procedures of the Official Methods of Analysis [17]. The nitrogen contents in the silage and concentrate were determined [17] and subsequently multiplied by a factor of 6.25 to obtain the crude protein content. The neutral detergent fiber (NDF), acid detergent fiber (ADF), and lignin contents were determined by the method of Van Soest et al. [18]; amylase was used for the NDF analyses of concentrate samples. The DM content in the silage was corrected using Equation (6), proposed by [19] to include the volatile solids in the DM.

*TDM* = 27.5 + 0.95 *ODM* (*p* < 0.001, *r*^2^ = 0.98) (6)

where *TDM* is the DM concentration determined by toluene distillation and *ODM* is DM concentration determined by oven drying.

The gross energy content of feces and feed offered was determined with an adiabatic bomb calorimeter (Parr Instrument Company, Moline, IL, USA).

### 2.9. Comparative Costs of the Respiration Chamber

The cost associated with the construction of the head-box respiration chamber described in the present paper is approximately 70% lower than a commercial chamber. This makes it suitable for developing countries where local engineers can build it using locally available materials. 

## 3. Results and Discussion

Table 3 shows the methane emission parameters measured in the head-box respiration chamber. The results show that the average volume of CH_4_ produced by high yielding Holstein Mexican dairy cows consuming 24.9 kg DM d^−1^ was 687 ± 123 L CH_4_ d^−1^, with a CH_4_ yield of 19.7 g kg^−1^ DMI, an emission intensity of 21.1 g kg^−^^1^ milk, and a *Ym* value of 5.7%. The average methane production for heifers was 248 L d^−1^ with a CH_4_ yield of 17.1 g kg^−1^ DMI and a *Ym* factor of 5.7%. Our results are in good agreement and within the range of methane emissions reported in recent studies. For example, Aubry and Yan [20] performed a meta-analysis using 987 individual observations obtained with respirometry chambers on cows of different ages, productivity stages, and breeds of dairy cattle in the United Kingdom (Holstein Friesian, Jersey × Holstein, and Norwegian). They reported a mean CH_4_ production of 467 ± 141 Ld^−1^ with a minimum of 141 L d^−1^ and a maximum production of 793 Ld^−1^ with a mean DMI of 14.8 kg DM d^−1^ (minimum 4.9 and maximum 26.1 kg DM d^−1^). So, the emission of our cows was similar to those cows with maximum emissions in the United Kingdom. However, a better comparison of CH_4_ emissions across studies can be achieved if methane yield (g of CH_4_ kg^−1^ DMI) is used instead of total daily CH_4_ production, because methane yield is a measure that is less affected by factors like the size of the animal, dry matter intake and, to a lesser extent, the type of diet. The CH_4_ yield (Table 3) obtained in the present work compares well with the average yield of 21.0 ± 0.45 g kg^−1^ DMI presented by Charmley et al. [2] who summarized data from 220 Australian dairy cows. Our CH_4_ yield for cows is also very close to the 19.2 g kg^−1^ of DMI reported by Appuhamy et al. [21] for dairy cows in North America, or the 19.1 g of CH_4_ kg^−1^ of DMI reported by Hristov et al. [3]. A more recent study conducted by Niu et al. [22], using 2566 individual records of emissions in order to develop intercontinental and regional-specific prediction equations for CH_4_ production, reported an average methane yield of 20.1 ± 3.87 g of CH_4_ kg^−1^ DMI. Similarly, van Lingen et al. [23] conducted a meta-analysis in order to develop prediction equations of enteric methane production using a transcontinental database with 1021 individual records of beef cattle emissions and reported an average methane yield of 20 ± 5.05 g of CH_4_ kg^−1^ DMI. So, the average methane yield obtained in the present study for cows is similar to those reported by all previous studies. Based on this published evidence and the recovery tests conducted in the present study, it is possible to say that the results of our respirometry system are correct and in line with most recent studies. The *Ym* factor obtained in our respiration chamber for dairy cows compares well with the *Ym*= 5.7% reported by Appuhamy et al. [21] for dairy cows in North America; it also agrees well with the Y*m*= 6.5% ± 1.0% suggested by [5] for cattle enteric CH_4_ inventory calculations, and with the average *Ym* of 6.0 presented by Niu et al. [22] and van Lingen et al. [23] in their transcontinental database for dairy and beef cattle, respectively. The mean CH_4_ yield intensity of 21.1 g of CH_4_ kg^−^^1^ milk observed in the present work is higher than the 13.5 g of CH_4_ kg^−^^1^ reported by Niu et al. [22]. This difference can be attributed to the higher milk yields observed in cows from the United States and the European Union; however, the intensity is within the range reported by Niu et al. [22], which goes from 3 to 36 g of CH_4_ kg^−^^1^ milk. 

Scarce information on CH_4_ production in young cattle is available. However, the results obtained here for heifers and calves are comparable with those of published studies. For example, Hammond et al. [24] conducted a study with Holstein and Friesian heifers of 14 months old with an average live weight of 339 ± 16 kg; they were fed twice daily (10:00 and 16:00 in equal amounts) with one of four conserved forage (haylage) treatments of ryegrass, clover trefoil and flowers in an experimental Latin square design (4 × 4). These authors found a CH_4_ production of 292 L d^−1^ for a DMI of 7.54 kg DM d^−1^, similar to the result obtained in the present study of 248 L CH_4_ d^−1^ for heifers with an average live weight of 339 ± 28 kg. Other studies by Jiao et al. [25,26] with Holstein heifers also revealed similar results: 225 L d^−1^ for 315-kg animals fed 2 kg d^−1^ of concentrate and grass silage ad libitum. Finally, it was reported by Pace et al. [12] that a limitation of using respiration chambers of the head-box type to measure CH_4_ emissions is that animals change their consumption behavior, thereby decreasing the production of CH_4_. However, in the present work, the observed consumption was similar to that reported in the literature for animals of equal weight. Furthermore, since the animals were accustomed to being in the head-box, they may have exhibited relatively normal behaviors.

## 4. Conclusions

The observed CH_4_ emissions and methane yield in the present study using a head-box type system are comparable to those reported by previous studies using similar measurement systems with open-circuit respiratory calorimeters. The head-box is a low-cost system that facilitates the accurate measurement of in vivo CH_4_ production from bovines and provides an option for countries with limited budget. 

## Figures and Tables

**Table 1:** Results of the full system recovery test.

Variable	Assay 1	Assay 2	Assay 3	Assay 4	Assay 5	Assay 6	Mean	Stand Dev.
Volume of air flow through the chamber, L/min	494.7	500.9	501.4	501.5	501.5	499.6	499.9	2.6
Duration of assay, min	30.9	59.0	22.9	21.0	19.2	103	42.9	
Volume of air through the chamber, m^3^	15.4	29.5	11.4	10.5	9.2	52.0	21.4	16.7
CH_4_ released, g	5.3	7.7	2.8	3.1	2	28.5	8.2	10.1
CH_4_ measured, g	5.2	8.2	3.0	3.2	2.0	28.5	8.4	
Ambient CH_4_ entering the chamber, %	0.0031	0.0018	0.0017	0.0017	0.0016	0.0010	0.0020	0.0006
Methane recovery rate	0.99	1.07	1.12	1.05	1.01	0.99	1.04	0.05

**Table 2:** Compositions of the silage and concentrate used in the experiment in g kg^−1^ DM determined by chemical analysis.

Variable	Heifers	Cows
Concentrate		
DM	885.3	888.5
OM	829.4	826.4
CP	219.4	221.1
NDF	326.4	340.7
ADF	94.5	123.5
Hemicellulose	231.9	217.2
Cellulose	51.3	83.3
Lignin	43.2	40.2
Silage		
DM	366.6	408.4
CP	121.8	114.7
NDF	487.2	511.5
ADF	368.5	377.6
Hemicellulose	118.7	135.4
Cellulose	261.5	314.2
Lignin	106.9	88.6
GE of the whole diet	17.0	16.7

DM = dry matter OM = Organic matter, CP = Crude protein, NDF = Neutral detergent fiber, ADF = Acid detergent fiber, GE = gross energy, MJ kg^−1^ DM.

**Table 3:** Mean in vivo CH_4_ production, dry matter and gross energy intakes, digestibility of dry matter and gross energy, methane conversion factor and methane yield for cows and heifers.

Variable	Cows	Stand. Dev.	95% Conf. Intervals	Heifers	Stand. Dev.	95% Conf. Intervals
	Mean			Mean		
Live weight, kg	593.4	50.9	512–674	338.5	27.8	294–382
DMI, kg d^−1^	24.9	0.5	24–25	10.4	0.5	9.6–11
DDM intake, kg d^−1^	18.6	1.1	16–20	6.5	0.7	5.3–7.5
Digestibility of diet, %	74.9	3.1	69–79	62.2	3.9	55–68
Gross energy intake, MJ d^−1^	424	8.5	16.9–17.1	173.0	7.8	160–185
Digestibility of gross energy, %	76.1	2.7	71–80	63.8	4.1	57–70
CH_4_ emission, L d^−1^	687	123	490–882	248.0	40.0	184–311
CH_4_ emission, g d^−1^	492	88.3	351–632	177.6	28.6	132–223
CH_4_, L kg^−1^ of DMI	27.6	4.8	19–35	24.0	4.8	16–31
CH_4_, L kg^−1^ DDM intake	36.7	5.3	28–45	39.0	9.8	23–54
CH_4_, g kg^−1^ of DMI	19.7	3.4	14–25	17.1	3.4	11.7–22.6
CH_4_, g kg^−1^ DDM intake	26.3	3.8	20–32	27.9	7.0	16–39
CH_4_, g kg^−1^ milk	21.1	3.3	15–26	-	-	-
*Ym*, %	6.4	1.1	4.6–8.1	5.7	1.1	3.8–7.5
Milk yield, kg d^−1^	23.3	1.8	20–26	-	-	-

DMI = Dry matter intake, DDM = digestible dry matter, Ym = methane conversion factor, energy of CH_4_ as a percentage of GEI; the specific energy of CHR_4_R is 55.65 MJ kg^−1^. DMI = Dry matter intake, MP = milk production, DDM = digestibility of dry matter, DOM = digestibility of organic matter, LMP = liters of milk produced, ± = standard deviation.
